# Energy Efficiency of Inference Algorithms for Clinical Laboratory Data Sets: Green Artificial Intelligence Study

**DOI:** 10.2196/28036

**Published:** 2022-01-25

**Authors:** Jia-Ruei Yu, Chun-Hsien Chen, Tsung-Wei Huang, Jang-Jih Lu, Chia-Ru Chung, Ting-Wei Lin, Min-Hsien Wu, Yi-Ju Tseng, Hsin-Yao Wang

**Affiliations:** 1 Department of Laboratory Medicine Chang Gung Memorial Hospital at Linkou Taoyuan City Taiwan; 2 Department of Information Management Chang Gung University Taoyuan City Taiwan; 3 Department of Electrical and Computer Engineering University of Utah Salt Lake City, UT United States; 4 Department of Computer Science and Information Engineering National Central University Taoyuan City Taiwan; 5 Graduate Institute of Biomedical Engineering Chang Gung University Taoyuan City Taiwan; 6 Department of Information Management National Central University Taoyuan City Taiwan

**Keywords:** medical informatics, machine learning, algorithms, energy consumption, artificial intelligence, energy efficient, medical domain, medical data sets, informatics

## Abstract

**Background:**

The use of artificial intelligence (AI) in the medical domain has attracted considerable research interest. Inference applications in the medical domain require energy-efficient AI models. In contrast to other types of data in visual AI, data from medical laboratories usually comprise features with strong signals. Numerous energy optimization techniques have been developed to relieve the burden on the hardware required to deploy a complex learning model. However, the energy efficiency levels of different AI models used for medical applications have not been studied.

**Objective:**

The aim of this study was to explore and compare the energy efficiency levels of commonly used machine learning algorithms—logistic regression (LR), k-nearest neighbor, support vector machine, random forest (RF), and extreme gradient boosting (XGB) algorithms, as well as four different variants of neural network (NN) algorithms—when applied to clinical laboratory datasets.

**Methods:**

We applied the aforementioned algorithms to two distinct clinical laboratory data sets: a mass spectrometry data set regarding *Staphylococcus aureus* for predicting methicillin resistance (3338 cases; 268 features) and a urinalysis data set for predicting *Trichomonas vaginalis* infection (839,164 cases; 9 features). We compared the performance of the nine inference algorithms in terms of accuracy, area under the receiver operating characteristic curve (AUROC), time consumption, and power consumption. The time and power consumption levels were determined using performance counter data from Intel Power Gadget 3.5.

**Results:**

The experimental results indicated that the RF and XGB algorithms achieved the two highest AUROC values for both data sets (84.7% and 83.9%, respectively, for the mass spectrometry data set; 91.1% and 91.4%, respectively, for the urinalysis data set). The XGB and LR algorithms exhibited the shortest inference time for both data sets (0.47 milliseconds for both in the mass spectrometry data set; 0.39 and 0.47 milliseconds, respectively, for the urinalysis data set). Compared with the RF algorithm, the XGB and LR algorithms exhibited a 45% and 53%-60% reduction in inference time for the mass spectrometry and urinalysis data sets, respectively. In terms of energy efficiency, the XGB algorithm exhibited the lowest power consumption for the mass spectrometry data set (9.42 Watts) and the LR algorithm exhibited the lowest power consumption for the urinalysis data set (9.98 Watts). Compared with a five-hidden-layer NN, the XGB and LR algorithms achieved 16%-24% and 9%-13% lower power consumption levels for the mass spectrometry and urinalysis data sets, respectively. In all experiments, the XGB algorithm exhibited the best performance in terms of accuracy, run time, and energy efficiency.

**Conclusions:**

The XGB algorithm achieved balanced performance levels in terms of AUROC, run time, and energy efficiency for the two clinical laboratory data sets. Considering the energy constraints in real-world scenarios, the XGB algorithm is ideal for medical AI applications.

## Introduction

Machine learning (ML) methods have been successfully employed in various medical fields [1-5], and energy consumption during ML inference has been attracting increasing attention [6-8]. The increasing focus on inference energy can primarily be attributed to two reasons. First, energy constraints constitute a major issue when ML is deployed into battery-powered medical devices [9-11]. Second, to achieve high predictive performance, the computation and memory requirements of ML models have increased. The growth of model size has been well reflected in neural networks (NNs) over the last decade, which are considered as the main ML algorithms implemented during this period.

An optimal ML model should achieve balanced predictive performance and energy efficiency. However, most relevant studies have only focused on comparing the predictive performance of different ML algorithms [12-14] and have not thoroughly explored the energy efficiency of different ML algorithms in the medical domain. Data formats in the medical field are diverse, and clinical laboratory data are a common type of medical data. In real-world settings, single laboratory tests must be subjected to strict validation procedures before their clinical use. Thus, the data obtained from such tests usually comprise features that are highly associated with the prediction targets. The characteristics of clinical laboratory data sets are unique, and the energy efficiency of different ML algorithms for processing clinical laboratory data sets warrants investigation.

A partial explanation for the poor understanding of energy efficiency is that estimating energy consumption is more difficult than estimating other metrics (eg, accuracy) [15]. Several methods exist for evaluating the energy consumption of ML models. Computational complexity can be used for theoretically approximating the number of operations; thus, it can be used to estimate energy consumption (Multimedia Appendix 1) [16-18]. Studies have established formulas for estimating energy consumption; these formulas sum the energy consumption levels of different elementary operations on the basis of complexity theory and benchmark results [7,19]. However, these formulas are available for only specific ML models and cannot be expanded to all algorithms. In addition to the aforementioned estimation formulas, experimental approaches can be used for estimating energy consumption. Currently, simulation and performance counters are the two main approaches for experimentally estimating energy consumption [15]. Although simulations enable fine-grained energy estimation at the architecture and instruction levels, the use of simulations for large-scale ML tasks is not feasible due to the considerable overhead involved [15]. By contrast, performance counters, which are a set of registers in processors that log specific hardware-related events, do not generate any overhead; therefore, these counters are suitable for use in different ML applications.

In this study, we estimated the power consumption of nine algorithms during ML inference: logistic regression (LR), k-nearest neighbor (kNN), support vector machine (SVM), random forest (RF), extreme gradient boosting (XGB), and four NN-based algorithms. These algorithms were used to classify two clinical laboratory data sets: a large binary feature set and a small integer feature set. The following performance measures were recorded: accuracy, area under the receiver operating characteristic curve (AUROC), time consumption, and power consumption. The time and power consumption were determined using performance counter data from Intel Power Gadget 3.5. Finally, we performed statistical tests to validate our results. The results indicated the energy efficiency of each investigated ML algorithm in medical applications.

## Methods

### Study Design and Environmental Settings

**Figure 1:**
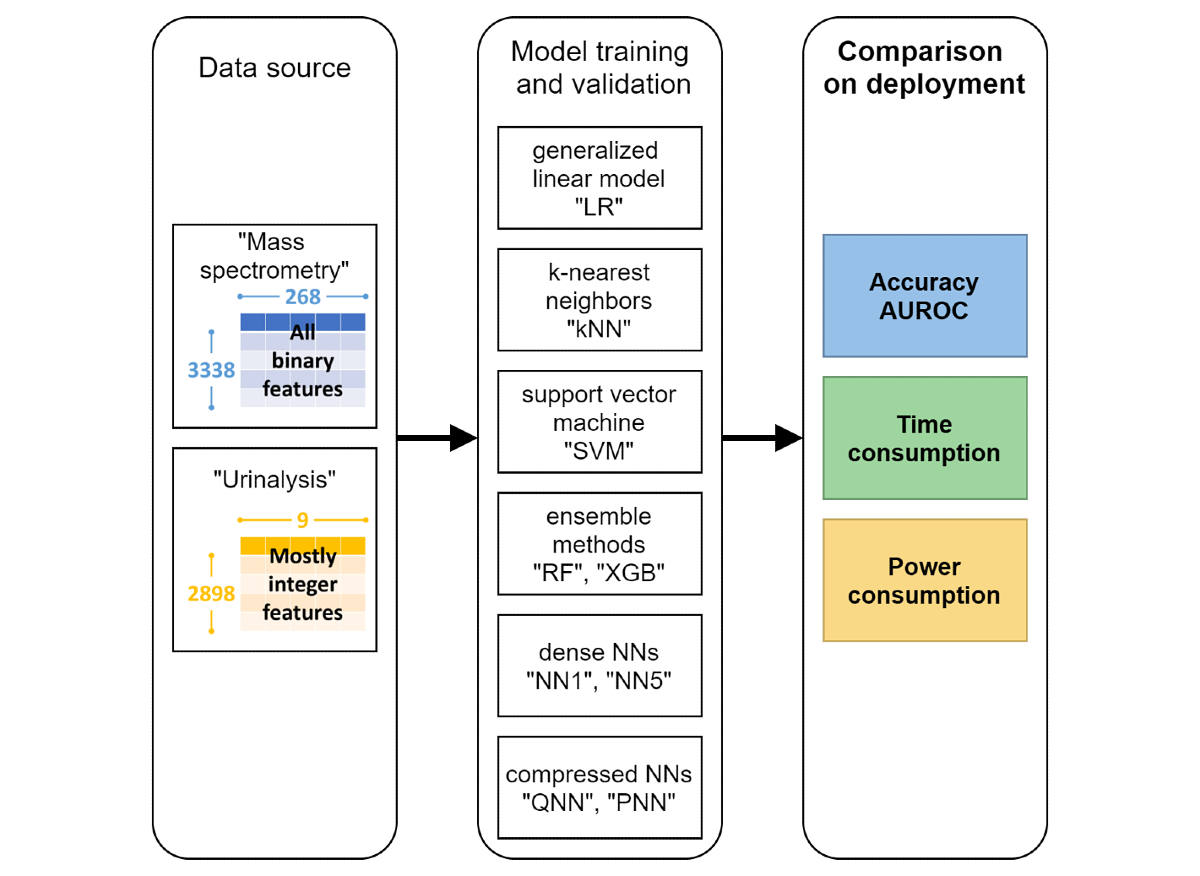
Process flowchart of this study. LR: logistic regression; kNN: k-nearest neighbor; SVM: support vector machine; RF: random forest; XGB: extreme gradient boosting; NN1: one-hidden-layer neural network; QNN: quantized five-hidden-layer neural network; PNN: pruned five-hidden-layer neural network; NN5: five-hidden-layer neural network; AUROC: area under the receiver operating characteristic curve.

Figure 1 illustrates the process flowchart of this study. We used two preprocessed data sets to train ML models with the nine considered algorithms: a mass spectrometry data set, based on matrix-assisted laser desorption/ionization time-of-flight mass spectrometry data of *Staphylococcus aureus* for predicting methicillin resistance, and a urinalysis data set, based on urinalysis data for predicting *Trichomonas vaginalis* infection. Subsequently, we comprehensively evaluated the trained models in terms of predictive performance, time consumption, and power consumption using independent testing data.

All experiments were run on a Windows 10 personal computer with 4 GB RAM and a 2.3 GHz Intel Core i5-8300H central processing unit (CPU). All ML models were implemented using Python 3.7.1 with the following Python libraries: scikit-learn 0.23.1 [20], xgboost 0.90 [21], and pytorch 1.8.1 [22]. Intel Power Gadget 3.5 [23] was used to acquire time and power measurements. Additional details regarding the power measurement and Intel Power Gadget 3.5 are provided in the “Time Consumption and Power Consumption” section below. All statistical analyses were performed using the “rstatix” package of R software (version 4.0.2).

### Data Source

#### Data Set Characteristics

The mass spectrometry and urinalysis data sets adopted in this study represent distinct feature patterns in ML; Table 1 presents their characteristics. The mass spectrometry data set has a relatively large feature set comprising 268 binary features. By contrast, the urinalysis data set has a small feature set comprising only nine features, almost all of which are integer features in a larger range. These data sets have been applied and validated in previous studies [24-26].

**Table 1:** Characteristics of the final mass spectrometry and urinalysis data sets.

Data set	Cases, n	Features, n	Binary features, n	Integer features, n	Majority class	Percentage of majority class (Nmax/N)	Gini Impurity
Mass spectrometry	3338	268	268	0	Methicillin-resistant *Staphylococcus aureus*	53.0%	0.50
Urinalysis	2898	9	2	7	*Trichomonas vaginalis*-negative	57.1%	0.49

#### Mass Spectrometry Data Set

The mass spectrometry data set comprises mass spectral information on methicillin-resistant and methicillin-sensitive *S. aureus* isolates. We collected routine mass spectrometry data about *S. aureus* samples consecutively from Chang Gung Memorial Hospital in 2016, and we identified the methicillin resistance of every *S. aureus* isolate by employing the paper disk method with cefoxitin.

In the original mass spectral data, intensity values are a function of the mass-to-charge ratio. We preprocessed these data using a validated binning method [24]. After data preprocessing, every feature in the mass spectrometry data set was determined to correspond to a 10-Da interval of mass-to-charge ratio. All the features are binary features, and the values 1 and 0 represent the presence and absence of a peak, respectively (ie, the presence and absence of a sufficient intensity, respectively), in a specific interval. The final size of the mass spectrometry data set was 3338×268 entries, with no missing values.

#### Urinalysis Data Set

The urinalysis data set comprises routine urinalysis data (including leukocyte esterase, nitrite, protein, occult blood, red blood cell count, white blood cell count, and epithelial cell count) and demographic data (age and sex) of patients with and without *T. vaginalis* infections. The diagnosis of *T. vaginalis* infection was made according to microscopic tests. The urinalysis data set comprises the data of all patients who received at least one urinalysis test at Chang Gung Memorial Hospital between January 2009 and December 2013. The original data set consists of 839,164 cases; because the outcome distribution is imbalanced in the original data [26], we applied random undersampling and the synthetic minority oversampling technique [27]. The final size of the urinalysis data set was 2898×9 entries and the percentage of the majority class was 57.1%.

In the urinalysis data set, “sex” and “nitrite” are binary features, which are represented by 1 and 0. “Leukocyte esterase,” “protein,” and “occult blood” data are semiquantitative features, which are represented on scales ranging from 0 to 4 (“negative,” “trace,” 1+, 2+, and 3+), 0 to 5 (“negative,” “trace,” 1+, 2+, 3+, and 4+), and 0 to 5 (“negative,” “trace,” 1+, 2+, 3+, and 4+), respectively. “Age,” “red blood cell count,” “white blood cell count,” and “epithelial cell count” are nonnegative integer features with maximum values of 103, 501, 501, and 101, respectively. The urinalysis data set does not contain missing values, and we did not perform further feature selection for this data set.

### Model Training and Validation

#### Algorithms

##### LR Algorithm

LR is one of the simplest binary classification algorithms. In the LR algorithm, the predictive outcome ŷ(**x**) of given data **x** is defined as follows:

ŷ(x)=[1+exp(w_0_+**w**^T^**x**)]^–1^

where **w** and **w_0_** represent the weight vector and bias of the LR model, respectively. LR is an example of a generalized linear model, and the output of the LR model represents the estimated probability of a certain class [17].

##### kNN Algorithm

The kNN algorithm is a memory-based algorithm. Accordingly, predictions of the kNN algorithm are directly based on the training data set and no additional training is required [28]. Predictions of the kNN algorithm, which are denoted by ŷ(**x**), are based on the voting results of the k most similar instances in the training dataset [29]. The parameter ŷ(**x**) is defined as follows:




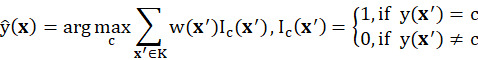




where K denotes the set of k instances in the training data set that are most similar to the given data **x** and **w(x_i_)** denotes the weighted value of the corresponding **x′** value. In the kNN algorithm, the distance between two data points **x** and **x′** is typically defined as follows:




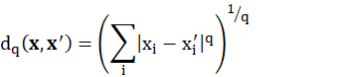




where q is a given positive number. The Manhattan distance (for q=1) and the Euclidean distance (for q=2) are the two most common distance metrics. In this study, the numbers of nearest neighbors (k) were 27 and 7 in the final models of the mass spectrometry and urinalysis data sets, respectively.

##### SVM Algorithm

The SVM algorithm is a commonly used binary classification method. The purpose of the SVM algorithm is to find a hyperplane that separates two classes of data with the maximum margin in the feature space [29]. In the original linear SVM, the output binary features are labeled as +1 and −1, and the predictive outcome ŷ(**x**) of given data **x** is defined as follows:

ŷ(**x**)=sign(w_0_+**w**^T^**x**)

where **w** and w_0_ represent the weight vector and bias of the SVM model, respectively.

The SVM algorithm is frequently applied with kernel transformations. Kernel functions represent the similarity between two data points. The radial basis function is one of the most commonly used kernel functions and is defined as follows:

κ(**x**,**x′**)=exp(–||**x**–**x′**||^2^/2*σ*^2^)

where *σ* is the bandwidth. Through kernel transformations, the original feature space can be mapped into a higher dimension, which may improve the predictive performance of the SVM algorithm. For a kernelized SVM algorithm, the following equation is obtained:




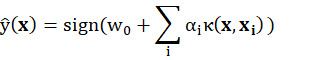




where **α** is a sparse vector. For all nonzero α_i_ values, corresponding **x_i_** terms represent support vectors. Accordingly, the final prediction ŷ(**x**) depends on only the support vectors and is independent of the remaining training data. In this study, we selected the kernelized SVM algorithm with the radial basis function kernel as our final SVM model according to its validation performance.

##### RF Algorithm

RF is an ensemble decision tree classifier. The prediction of the RF algorithm, namely ŷ(**x**), depends on the voting results of numerous decision trees [12]. The parameter ŷ(**x**) is defined as follows:




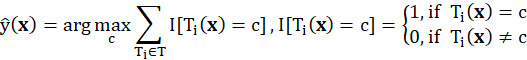




where T represents the set of decision trees in the RF and T_i_(**x**) represents the predictive outcome of a given decision tree.

RF training involves the “bagging” (ie, bootstrap aggregating) technique [30]. Accordingly, each decision tree in the RF considers only a subset of training cases to improve model generalizability. In addition to the bagging technique, each split of the decision trees only considers a subset of the input features during training to prevent the growth of highly correlated trees [31]. In this study, the number of trees was set to 1000 and 1500 in the final RF models for the mass spectrometry and urinalysis data sets, respectively.

##### XGB Algorithm

The XGB algorithm is a type of ensemble algorithm, which uses the “boosting” technique to reduce the overall bias by sequentially combining weak classifiers into a model [32]. In practice, shallow decision trees are typical weak classifiers. The outcome ŷ(**x**) of XGB models represents the log odds ratio of a certain class in binary classification tasks. The parameter ŷ(**x**) is defined as follows:




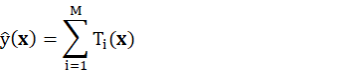




where T_i_(**x**) denotes the *M* decision tree regressors in the model.

In each iteration, the training of decision trees in XGB is equivalent to a process of minimizing a certain objective function. Because XGB is a regularized algorithm [21], its objective function is different from those of the original gradient boosting algorithms. The objective function of a given decision tree in the XGB algorithm can be expressed as follows:




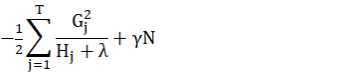




where N denotes the number of leaf nodes in a decision tree and γ and λ denote given positive numbers. The parameters G_j_ and H_j_ are defined as follows:




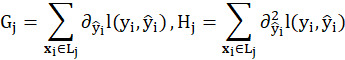




where ŷ_i_ represents the predictive outcome of training data **x_i_** after a certain number of iterations, l(y_i_,ŷ_i_) represents a certain loss function between the predictive and actual outcomes, and L_j_ represents the set of data points **x**_i_ belonging to the *j*th leaf node.

In this study, we implemented the XGB model by using the “xgboost 0.90” Python library.

##### NN Algorithms

NN is a type of ML model that is inspired by the human nervous system. An NN consists of multiple layers of nodes (or neurons). The layer that receives the initial data is the input layer and the layer that exports the predictive results is the output layer. Numerous hidden layers exist between the input and output layers. The outputs of each node in an NN are obtained according to the outputs of the nodes in the previous layer [12].

Several types of connection patterns are possible between two adjacent layers. For example, in a classic fully connected layer, a series of weighted sums of the inputs is first calculated according to the given model parameters. These weighted sums are subjected to nonlinear transformation to obtain the output of the aforementioned layer. In practice, these steps are implemented using vectorized expressions, and the output vector of the *n*th hidden layer, namely **a_n+1_,** is expressed as follows:

**a_n+1_**=g(**Θ_n_**·**a_n_**+**b_n_**)

where **a_n_**, **Θ_n_**, and **b_n_** represent the input vector, weight matrix, and bias vector of the hidden layer, respectively, and g represents a nonlinear activation function (eg, the sigmoid function or rectified linear unit activation function).

NNs are ML models that are flexible in terms of the numbers of hidden layers and nodes in each layer. According to previous studies, one hidden layer is sufficient for approximating most continuous functions [33,34]. By contrast, NNs with more than one hidden layer are called deep NNs, and they have superior generalization ability to one-hidden-layer NNs [35,36]. With improvement of the hardware, deep learning models have become increasingly popular over the past few years. In this study, we constructed two types of NNs, namely a one-hidden-layer NN (NN1) and a five-hidden-layer NN (NN5), as our underlying architectures. NN1 represents the simplest form of NNs, whereas NN5 represents a deep learning model.

To determine the appropriate architecture of an NN model, some previous studies offered theoretical heuristics regarding the number of hidden units in an NN layer. However, the results ranged widely according to different studies regarding the optimal number of nodes in a hidden layer [37-40]. Accordingly, in this study, we selected the final number of hidden units according to the cross-validation results. After hyperparameter tuning, we determined that the final sizes of the NN1 and NN5 architectures were 268×2048×1 and 268×1024×1024×1024×1024×1024×1, respectively, for the mass spectrometry data set and 9×128×1 and 9×512×512×512×512×1, respectively, for the urinalysis data set.

##### Pruned NNs

Pruning is a method for eliminating redundant connections in NNs [41]. In this method, an NN is converted into a sparse model to reduce its size. Pruning methods can be unstructured or structured [42]. Unstructured pruning eliminates the individual parameters in an NN, whereas structured pruning eliminates the connections in large units such as hidden units in a fully connected layer or channels in a convolutional layer.

In this study, we applied global unstructured pruning to eliminate connections from the entire NN. The pruned NNs displayed in the figures are NN5s with a sparsity of 50%. However, we implemented pruning with sparsity values of 25%, 50%, and 75% for the NN1 and NN5 models. The detailed results regarding other pruned NNs are provided in Multimedia Appendix 2-5.

##### Quantized NNs

Quantization is a common method for model compression. In this method, the model size is reduced by computing and storing parameters with low bit widths [43]. Two main quantization methods exist in the Pytorch framework: dynamic and static quantization [22]. Dynamic quantization is the simplest quantization method. In dynamic quantization, the weights of the quantized layers in an NN are replaced with low-precision data, and the activations are quantized just before entering each quantized layer during inference. By contrast, in static quantization, the parameters for activation quantization are determined before the inference phase. Therefore, static quantization requires an additional calibration with a data set before inference.

In this study, the parameters of the original NN1 and NN5 models were tensors in the single-precision floating-point format; the quantized models had a quantized 8-bit signed integer data format. The quantized NNs displayed in the figures are NN5s. However, we implemented dynamic quantization for both the NN1 and NN5 models, and the detailed results regarding quantized NNs are provided in Multimedia Appendix 2-5.

#### Model Construction

In this study, we selected the aforementioned supervised ML algorithms according to their maturity and popularity. For every ML model, we tuned the hyperparameters in each algorithm through 5-fold cross-validation. The cutoff with the highest Youden index was selected as the final cutoff in each model [44].

### Model Comparison on Deployment

#### Predictive Performance

We evaluated the predictive performance of all final models using independent testing data sets. We selected accuracy and AUROC as the predictive performance metrics. The 95% CIs of both accuracy and AUROC were calculated.

#### Time and Power Consumption

We derived the inference time and power data from Intel Power Gadget 3.5 [23]. This commercial product provides power data on the basis of Intel Running Average Power Limit (RAPL) interface estimation. RAPL is a driver that provides a set of performance counter data on time, power, and energy [45,46].

We implemented the ML models using command lines and logged the time and power data using PowerLog3.0.exe, a command line version of Intel Power Gadget that allows users to log the time and power data of a specific command line. In addition, because Intel Power Gadget only provides the energy data of the entire processor, all testing procedures were performed without background programs. The measurement for each algorithm was repeated 100 times.

#### Statistical Analysis

We initially employed the Shapiro-Wilk test to check for normality. If the assumption of normality did not hold, we subsequently adopted the Friedman test to compare the means of different groups. The pairwise Wilcoxon signed-rank test was used to identify which groups were different. *P* values were adjusted using the Bonferroni multiple testing correction method. All statistical tests were two-sided with an α error level of .05.

## Results

### Predictive Performance of ML Algorithms

**Figure 2:**
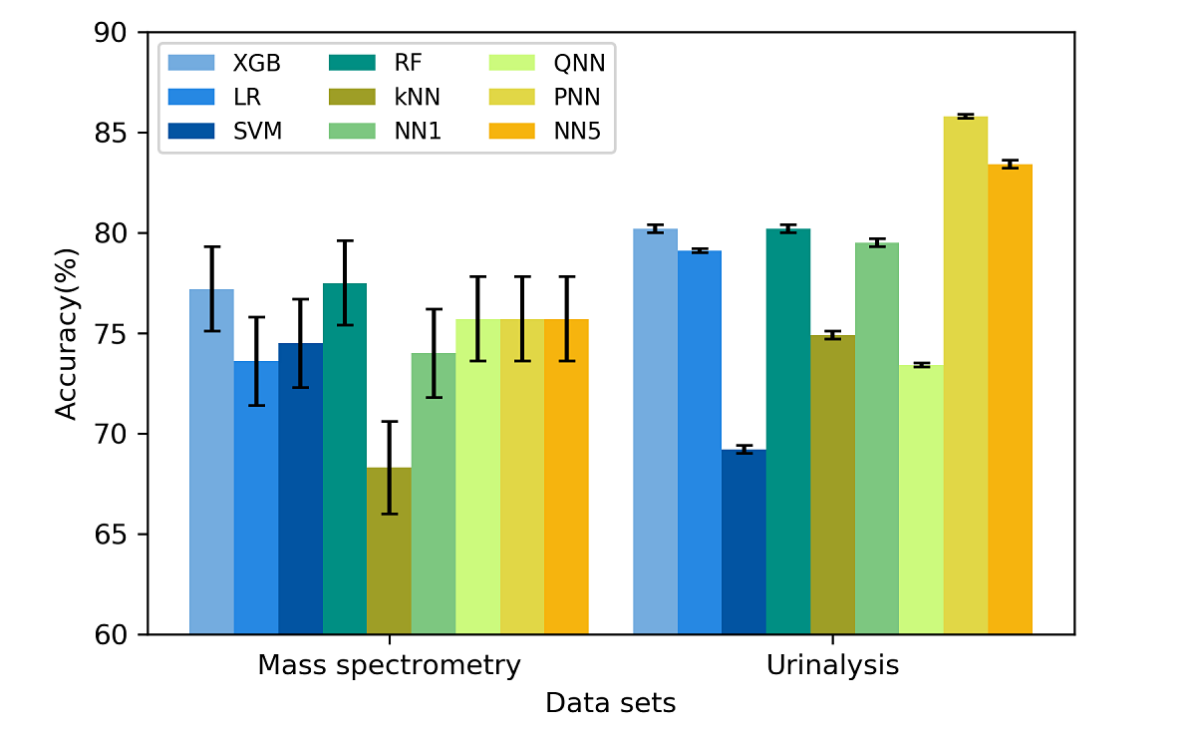
Classification accuracy rates of different algorithms implemented on the mass spectrometry and urinalysis data sets. The black bars indicate the 95% CIs of the classification accuracy. LR: logistic regression; kNN: k-nearest neighbor; SVM: support vector machine; RF: random forest; XGB: extreme gradient boosting; NN1: one-hidden-layer neural network; QNN: quantized five-hidden-layer neural network; PNN: pruned five-hidden-layer neural network; NN5: five-hidden-layer neural network.

**Figure 3:**
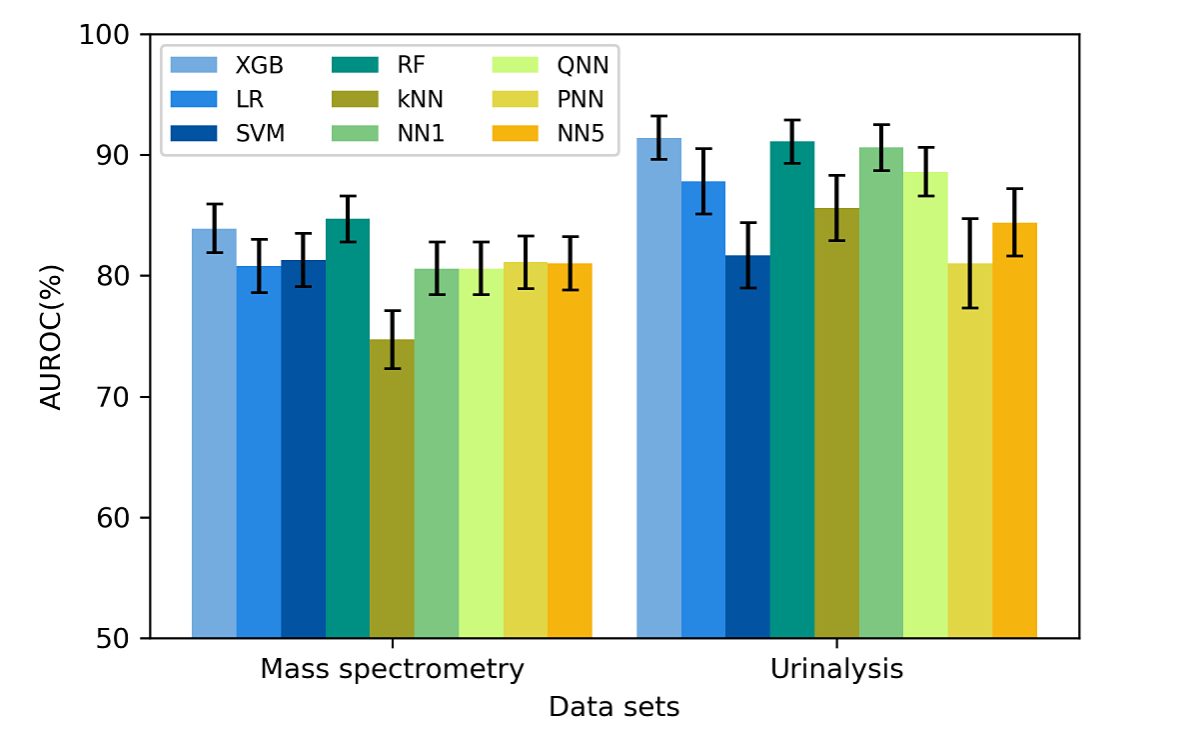
AUROC values of different algorithms implemented on the mass spectrometry and urinalysis data sets. The black bars indicate the 95% CIs of the AUROC. LR: logistic regression; kNN: k-nearest neighbor; SVM: support vector machine; RF: random forest; XGB: extreme gradient boosting; NN1: one-hidden-layer neural network; QNN: quantized five-hidden-layer neural network; PNN: pruned five-hidden-layer neural network; NN5: five-hidden-layer neural network; AUROC: area under the receiver operating characteristic curve.

Figure 2 and Figure 3 display the classification accuracy rates and AUROC values for the various ML models, respectively. Almost all models had high accuracy rates. All algorithms, except for the kNN algorithm, achieved an accuracy rate of at least 70% for the mass spectrometry data set. Moreover, all algorithms, except for the SVM algorithm, achieved an accuracy rate of at least 70% for the urinalysis data set. As displayed in Figure 3, the two tree-based methods, namely the RF and XGB algorithms, achieved the two highest AUROC values for both datasets (84.7% and 83.9% for the mass spectrometry data set, respectively; 91.1% and 91.4% for the urinalysis data set, respectively). In particular, the RF and XGB algorithms exhibited significantly higher AUROC values than those of most of the other algorithms (eg, kNN, SVM, pruned five-hidden layer NN [PNN], and NN5) for the urinalysis data set. The results regarding the algorithms’ predictive performance are detailed in Multimedia Appendix 2 and Multimedia Appendix 3.

### Inference Times of ML Algorithms

**Figure 4:**
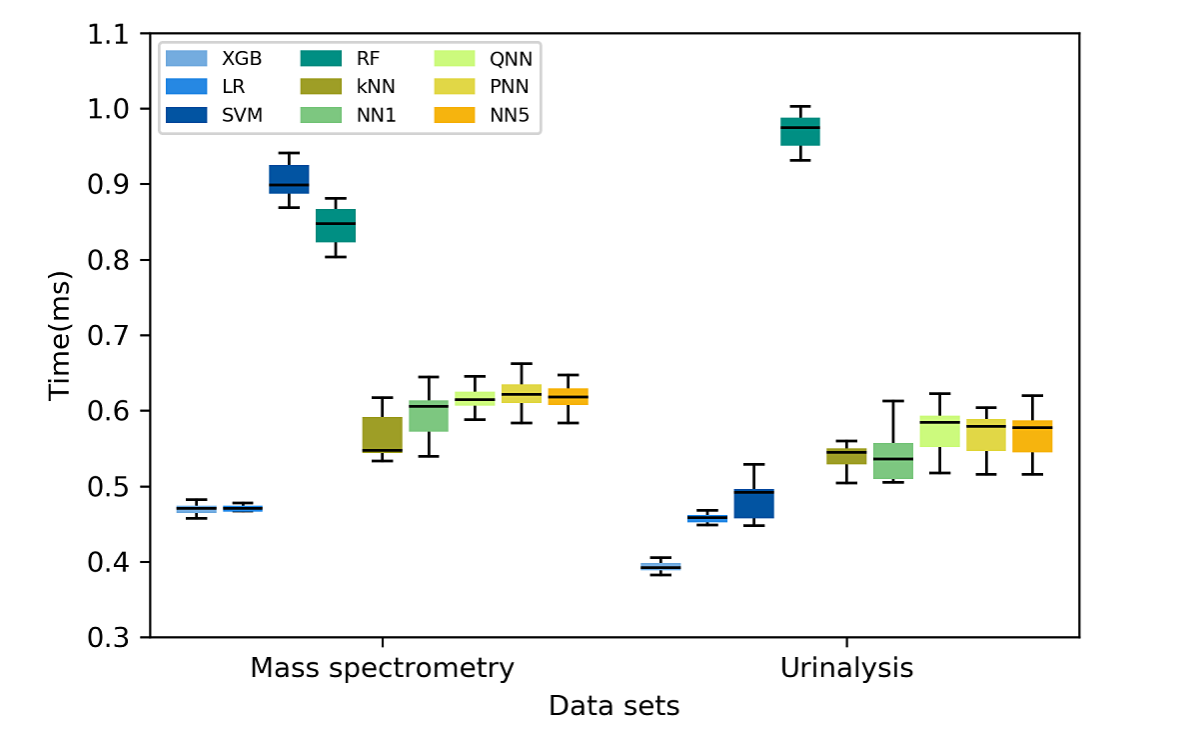
Time consumed in single prediction for the mass spectrometry and urinalysis data sets. LR: logistic regression; kNN: k-nearest neighbor; SVM: support vector machine; RF: random forest; XGB: extreme gradient boosting; NN1: one-hidden-layer neural network; QNN: quantized five-hidden-layer neural network; PNN: pruned five-hidden-layer neural network; NN5: five-hidden-layer neural network.

Figure 4 presents a comparison of the inference times of the various ML algorithms. All algorithms completed the inference process within 1 millisecond. The XGB and LR algorithms had the shortest runtimes (0.47 milliseconds for both in the mass spectrometry data set; 0.39 and 0.47 milliseconds, respectively, for the urinalysis data set). The Wilcoxon signed-rank test results revealed that the run times of these two algorithms differed significantly (*P*<.001) from those of the other algorithms, except for NN1. The SVM and RF algorithms exhibited the highest time consumption for the mass spectrometry and urinalysis data sets, respectively. In particular, the RF algorithm exhibited a higher run time compared with that of all other algorithms, except for the SVM algorithm, for both data sets (*P*<.001). The results regarding the time consumption of the algorithms are detailed in Multimedia Appendix 4-5, and the corresponding *P* values derived from the Wilcoxon signed-rank test are presented in Multimedia Appendix 6-7.

### Power Consumption of ML Algorithms

**Figure 5:**
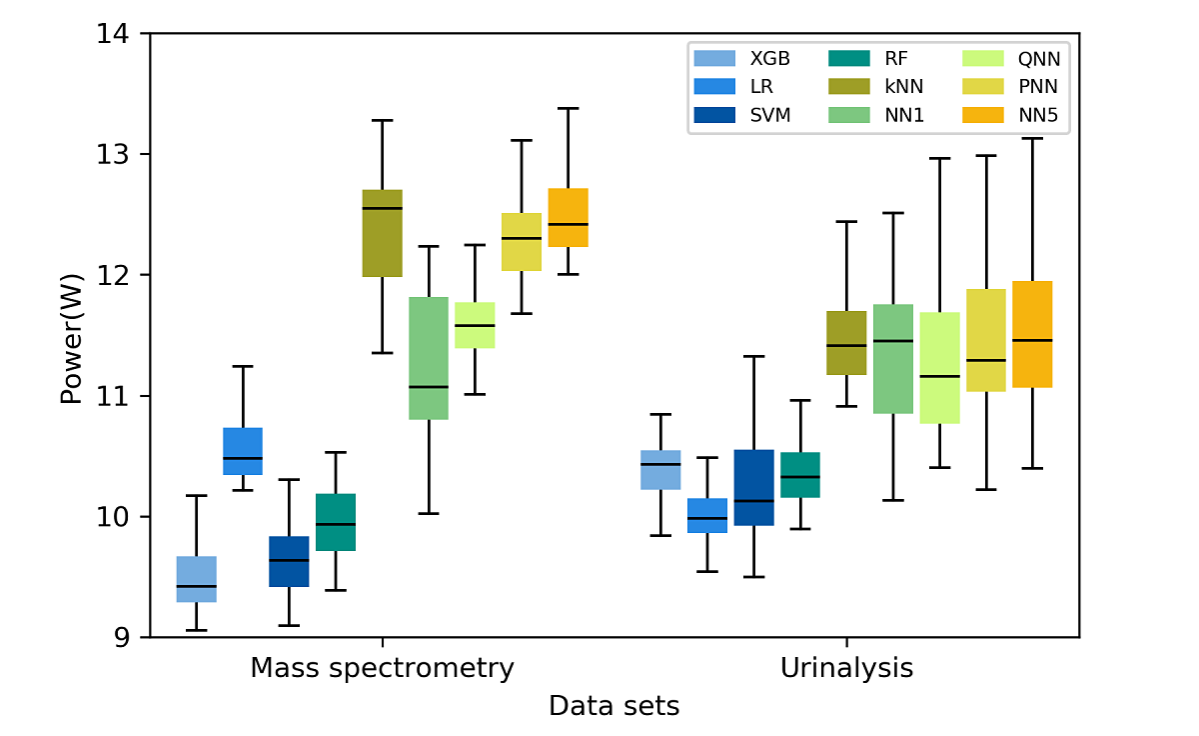
Power consumption levels of the different algorithms implemented on the mass spectrometry and urinalysis data sets. LR: logistic regression; kNN: k-nearest neighbor; SVM: support vector machine; RF: random forest; XGB: extreme gradient boosting; NN1: one-hidden-layer neural network; QNN: quantized five-hidden-layer neural network; PNN: pruned five-hidden-layer neural network; NN5: five-hidden-layer neural network.

Figure 5 presents a comparison of the power consumption levels of the ML algorithms. Algorithms of the same type consumed similar amounts of power. For example, both tree-based algorithms (RF and XGB) consumed limited power, whereas all NN-based models (NN1, quantized five-layer hidden NN [QNN], PNN, and NN5) consumed considerable power. The XGB algorithm exhibited the lowest power consumption for the mass spectrometry data set (9.42 Watts) and the LR algorithm exhibited the lowest power consumption for the urinalysis data set (9.98 Watts). According to the results of the Wilcoxon signed-rank tests (Multimedia Appendix 6-7), the LR and XGB algorithms exhibited lower power consumption levels than did the kNN algorithm and all NN-based algorithms for both datasets (*P≤*.001). The NN5, kNN, PNN, QNN, and NN1 algorithms exhibited higher power consumption levels compared with those of the other algorithms. Although pruning and quantization reduced the power consumption levels of the NN algorithms, the energy efficiency levels of the PNN and QNN algorithms did not surpass those of all the non-NN–based algorithms, except for the kNN algorithm. The results regarding power consumption are detailed in Multimedia Appendix 4-5, and the corresponding *P* values derived from the Wilcoxon signed-rank test are presented in Multimedia Appendix 6-7.

### Overall Comparison

**Figure 6:**
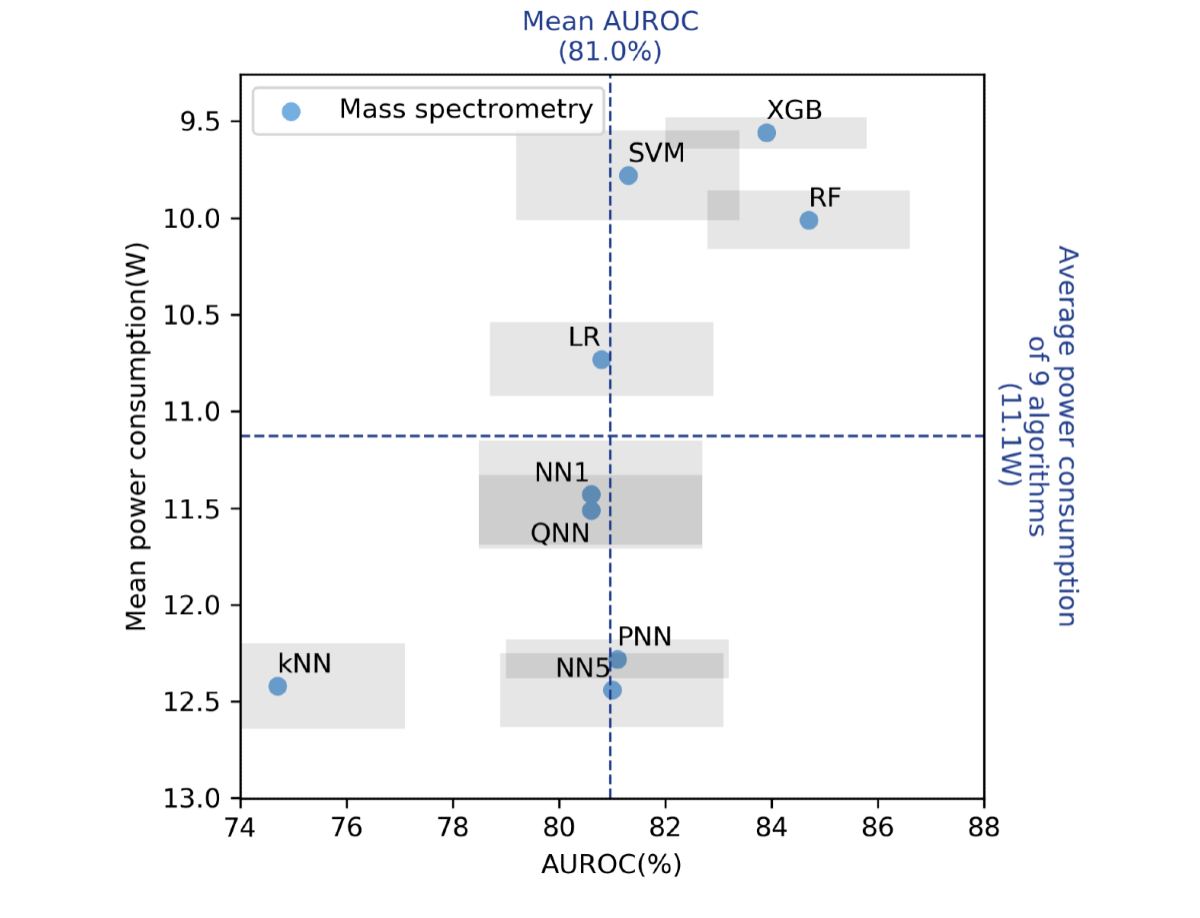
Predictive performance (AUROC)–power consumption plot of the nine algorithms for the mass spectrometry data set. The two tree-based algorithms (RF and XGB) achieved a balanced predictive performance and power consumption. The horizontal and vertical dashed axes indicate the mean energy consumption and mean AUROC of the nine predictive models, respectively. Each algorithm is located in one of the four quadrants. The gray rectangle around each data point denotes the 95% CI of the AUROC and power consumption. LR: logistic regression; kNN: k-nearest neighbor; SVM: support vector machine; RF: random forest; XGB: extreme gradient boosting; NN1: one-hidden-layer neural network; QNN: quantized five-hidden-layer neural network; PNN: pruned five-hidden-layer neural network; NN5: five-hidden-layer neural network; AUROC: area under the receiver operating characteristic curve.

**Figure 7:**
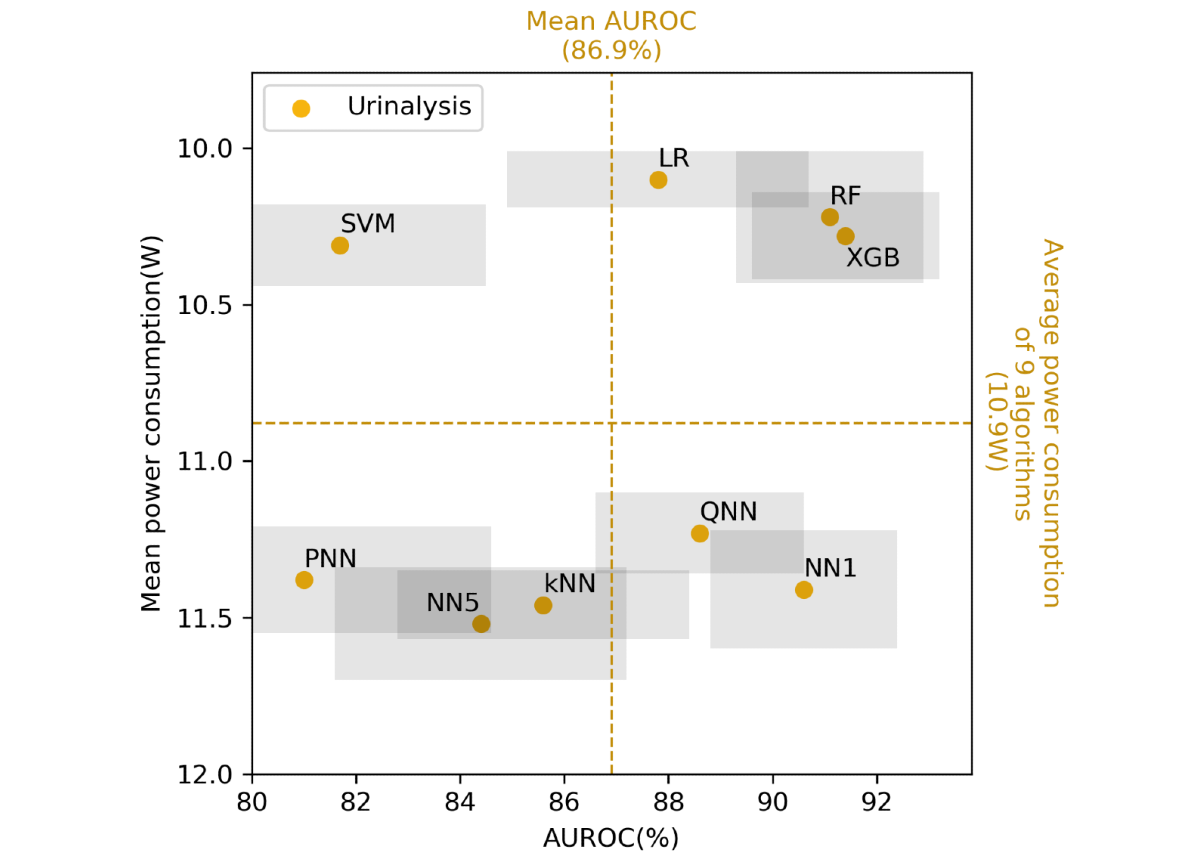
Predictive performance (AUROC)–power consumption plot of the nine algorithms for the urinalysis dataset. The two tree-based algorithms (ie, RF and XGB) achieved a balanced predictive performance and power consumption. The horizontal and vertical dashed axes indicate the mean energy consumption and mean AUROC of the nine predictive models, respectively. Each algorithm is located in one of the four quadrants. The gray rectangle around each data point denotes the 95% CI of the AUROC and power consumption. LR: logistic regression; kNN: k-nearest neighbor; SVM: support vector machine; RF: random forest; XGB: extreme gradient boosting; NN1: one-hidden-layer neural network; QNN: quantized five-hidden-layer neural network; PNN: pruned five-hidden-layer neural network; NN5: five-hidden-layer neural network; AUROC: area under the receiver operating characteristic curve.

Figure 6 and Figure 7 display scatter plots of the performance of the various algorithms in predicting *S. aureus* methicillin resistance and *T. vaginalis* infection. The horizontal and vertical axes in these figures represent the AUROC and average power consumption, respectively. The two dashed lines in the figures represent the average AUROC values and mean power consumption levels for the nine algorithms. Only the XGB and RF algorithms had higher than average AUROC and power consumption results for both data sets. Figure 6 and Figure 7 also illustrate the difference between the NN-based and non-NN–based algorithms. All NN-based algorithms are located in the lower half-plane in these figures and all the other algorithms, except for the kNN algorithm, are located in the upper half-plane in the figures. These results indicate that the NN-based algorithms had higher power consumption levels than those of the non-NN–based algorithms, even when model compression was executed through methods such as pruning or quantization.

## Discussion

### Principal Findings and Related Works

In this study, we compared the predictive performance, time consumption, and power consumption of nine algorithms using two clinical laboratory data sets. The XGB algorithm achieved a balanced performance with respect to the aforementioned metrics, indicating that the XGB algorithm is ideal for medical artificial intelligence applications with energy constraints.

In addition to this study, previous studies have performed comparative analyses of various ML algorithms in the medical domain [12,13,47]. However, only few studies have considered the inference efficiency in addition to the predictive performance. Zhang et al [13] compared the simplicity of seven algorithms by assessing their memory usage and training time for 12 public biomedical data sets. In another study, Deng et al [47] assessed the inference time of decision tree, SVM, RF, and NN algorithms. In this study, we executed our efficiency evaluation by directly exploring and comparing the power consumption levels of ML algorithms. Furthermore, all power consumption data were obtained according to real-time experimental results from performance counters.

### Predictive Performance of ML Algorithms

The RF and XGB algorithms exhibited higher AUROC values than did the other algorithms for both data sets. This finding is similar to those of previous studies. In a study that considered 11 performance metrics, the RF algorithm and probability-calibrated boosted trees exhibited the best performance among 10 algorithms [48]. Other previous analyses also indicated that the RF and XGB algorithms consistently exhibit good performance for most biomedical data sets [13,14]. These algorithms have certain advantages; for example, they exhibit adequate scalability to large data sets and are more robust than other types of algorithms [17]. Medical data sets usually comprise features with strong signals; this is because only well-validated markers are routinely tested in clinical scenarios. Under this condition, tree-based methods would not be inferior to relatively complex models such as NN-based models. However, one should remember the “no free lunch theorem” [49], which suggests that no model exhibits superior performance universally. This statement is true because every algorithm is proposed on the basis of different underlying assumptions, which may fit only specific types of data. Therefore, different algorithms should be investigated when the predictive performance of a certain model does not match the expectation.

### Inference Time of ML Algorithms

In this study, the XGB and LR algorithms exhibited the shortest run times (both 0.47 milliseconds for the mass spectrometry data set; 0.39 and 0.47 milliseconds, respectively, for the urinalysis data set). The SVM and RF algorithms exhibited the highest time consumption levels for the mass spectrometry and urinalysis data sets, respectively. Notably, although the XGB and RF algorithms are ensemble algorithms based on decision trees, the XGB algorithm consumed less time than the RF algorithm. This finding is possibly due to differences in the depth and number of trees between these algorithms. For both data sets, the XGB model had shallower trees than did the RF model (for the mass spectrometry and urinalysis data sets, the maximum depths of the XGB decision trees were 6 and 10, respectively, and the average depths of the RF trees were 29 and 21, respectively). An explanation for this finding is that boosting reduces the bias of weak classifiers [17,50] and that bagging reduces the variance of complex classifiers [51]. Thus, the XGB algorithm may have shallower decision trees compared with those of the RF algorithm for the same prediction task. In addition to the depth difference, the number of trees may be another cause of the run time difference between the two tree-based algorithms (for the mass spectrometry and urinalysis data sets, the XGB algorithm contained 120 and 32 decision trees, respectively, and the RF algorithm contained more than 1000 decision trees). In an RF model, increasing the number of decision trees does not engender overfitting [18,30]. However, this characteristic may result in a final model with excessive decision trees after conventional grid-search cross-validation. By contrast, because an excessive number of decision trees results in overfitting in an XGB model, an XGB model with optimal predictive performance would have an appropriate number of trees. Furthermore, to identify the suitable tree numbers, the early stopping technique is frequently used during training of XGB models in practice [52]. In conclusion, the shorter run time of the XGB algorithm compared with the RF algorithm is possibly due to the different characteristics of these algorithms.

### Power Consumption of ML Algorithms

The NN algorithms (NN1, QNN, PNN, and NN5) and the kNN algorithm exhibited the highest power consumption levels in this study, and the two tree-based algorithms (ie, RF and XGB) exhibited the lowest power consumption levels. Tree-based algorithms use the data structure of search trees for making inferences. The inference process mainly involves comparison operations at tree nodes and irregular memory access operations for subtree retrievals. In contrast to several other ML algorithms, tree-based algorithms typically do not use multiplication operations. The comparison and memory access operations in tree-based algorithms consume less energy than do multiplication operations [53,54]. The experiments in this study were run on a general-purpose CPU. Therefore, if necessary, the energy efficiency of tree-based algorithms can be increased using specialized hardware accelerations [55-57].

NNs have been regarded as the main tools for implementing ML in the last few years. The development of different NN architectures (eg, convolutional NNs and recurrent NNs) has contributed to considerable improvements in unstructured data analyses [35,55]. However, NNs have high power consumption. Thus, NNs should not always be considered as the preferred algorithm for implementing ML, unless they exhibit superior predictive performance compared with other algorithms. In this study, the adopted NNs consumed considerable power because of their high computational and communication demands. The computational demand of an NN refers to the large number of multiply-add operations in the forward propagation process, and the communicational demand of an NN refers to the energy cost of moving large quantities of data frequently between the processor and memory [7,58].

Several methods are available for reducing the power consumption of NNs. NNs have diverse architectures, and constructing an NN with a small architecture is an effective method for improving energy efficiency, as reflected by the difference in power consumption between the NN1 and NN5 models in this study (see Multimedia Appendix 3 and 5). In addition to constructing a small model, a given NN model can be compressed to reduce power consumption. In this study, we implemented and evaluated two common methods for NN compression, namely pruning [10,41] and quantization [59]. According to the obtained results, these model compression methods reduced the power consumption levels of the NNs. However, the NN-based algorithms did not exhibit higher energy efficiency levels compared with those of the non-NN–based algorithms, even after model compression. Furthermore, although energy optimization methods such as quantization are frequently used for NNs, these methods are not specific to NNs [60,61]. Thus, quantization can be feasibly applied to other ML algorithms if their power consumption must be decreased.

### Overall Comparison

In summary, the XGB algorithm achieved balanced predictive performance and energy efficiency levels. Figure 6 and Figure 7 display the predictive performance–power consumption plots of the nine algorithms for the mass spectrometry and urinalysis data sets, respectively. In these figures, the two tree-based algorithms, namely the XGB and RF algorithms, are located in the right-upper quadrant, which indicates that they had higher than average predictive performance and lower than average power consumption. However, the XGB algorithm consumed less time than the RF algorithm (*P<*.001, according to the Wilcoxon signed-rank test; Figure 4 and Multimedia Appendix 6-7). Thus, the XGB algorithm achieved a higher energy efficiency level than the RF algorithm because the overall energy consumption for ML inference depends on not only power consumption but also on inference time.

Deep learning models are the main ML algorithms applied currently. These algorithms achieve state-of-the-art predictive performance for unstructured data sets (eg, data sets for computer vision and natural language processing) [55]. However, deep learning algorithms may be unnecessary for making predictions based on clinical laboratory data sets. In Figures 6 and 7, all of the NN-based algorithms are located in the lower half-plane, signifying that the NN-based algorithms consumed more power than did most of the other algorithms. Pruning and quantization increased the efficiency levels of the NN-based algorithms; however, the increase was limited, and the energy efficiency levels of these algorithms did not surpass that of the XGB algorithm. Moreover, the NN-based algorithms did not exhibit higher AUROC values compared with those of the simple tree-based algorithms. The experimental results indicate that for data analysis in the clinical laboratory domain, simpler models such as the XGB model may be sufficient to achieve state-of-the-art predictive performance. Deep NNs are unsuitable for such data sets due to the high power consumption of these networks.

### Limitations

This study has some limitations. First, because Intel Power Gadget 3.5 only provides the energy consumption of the entire processor [15], one should focus on the comparison of the investigated ML algorithms and not on the absolute power consumption obtained. Second, this study considered only two clinical laboratory data sets. Because energy consumption varies between data sets, a large-scale study based on a variety of medical data sets is essential for confirming the results of this study. Finally, the results were obtained using a general-purpose CPU; however, energy consumption may vary across different processors. Currently, ML is frequently implemented using hardware acceleration techniques. Although hardware devices such as discrete graph processing units or tensor processing units are not ubiquitous equipment in clinical settings, their energy efficiency levels are worth investigation. Energy efficiency is a major issue in embedded systems, and studies have been performed on the energy optimization of different algorithms [6,11,19]. Executing a fair comparison of energy efficiency under different hardware implementations is difficult. Hence, a well-designed comparative analysis of energy efficiency across different optimized methods is essential for obtaining general conclusions.

### Conclusions

This study comprehensively compared various ML algorithms in terms of their predictive performance, time consumption, and power consumption when implemented on two clinical laboratory data sets. According to the results, the XGB algorithm attained balanced performance levels in terms of the aforementioned parameters for the two data sets. Thus, the XGB algorithm is ideal for application in real-world clinical settings.

## Appendix

Multimedia Appendix 1 Time complexity of some common algorithms.

Multimedia Appendix 2 Classification performance of nonneural network–based machine learning algorithms implemented on the mass spectrometry and urinalysis data sets.

Multimedia Appendix 3 Classification performance of different neural networks (NNs) implemented on the mass spectrometry and urinalysis data sets.

Multimedia Appendix 4 Inferencing time and average power consumption levels of nonneural network–based algorithms implemented on the mass spectrometry and urinalysis data sets.

Multimedia Appendix 5 Inferencing time and average power consumption levels of different neural networks (NNs) implemented on the mass spectrometry and urinalysis data sets.

Multimedia Appendix 6 *P* values were derived from the pairwise Wilcoxon signed-rank test to identify which time and power consumption of any two algorithms were different on the mass spectrometry data set. The *P* values were adjusted by the Bonferroni multiple testing correction method. LR, logistic regression; kNN, k-nearest neighbors; SVM, support vector machine; RF, random forest; XGB, extreme gradient boosting; NN1, one-hidden-layer neural network; QNN, quantized five-hidden-layer neural network; PNN, pruned five-hidden-layer neural network; NN5, five-hidden-layer neural network.

Multimedia Appendix 7 *P* values were derived from the pairwise Wilcoxon signed-rank test to identify which time and power consumption of any two algorithms were different on the Urinalysis dataset. The adjusted *P* values was adjusted by the Bonferroni multiple testing correction method. LR, logistic regression; kNN, k-nearest neighbors; SVM, support vector machine; RF, random forest; XGB, extreme gradient boosting; NN1, one-hidden-layer neural network; QNN, quantized five-hidden-layer neural network; PNN, pruned five-hidden-layer neural network; NN5, five-hidden-layer neural network.

## Abbreviations

AUROC: area under the receiver operating characteristic curve
CPU: central processing unit
kNN: k-nearest neighbor
LR: logistic regression
ML: machine learning
NN: neural network
NN1: one-hidden-layer neural network
NN5: five-hidden-layer neural network
PNN: pruned five-hidden-layer neural network
QNN: quantized five-hidden-layer neural network
RAPL: Running Average Power Limit
RF: random forest
SVM: support vector machine
XGB: extreme gradient boosting
