# Supplementary material for: Energy Efficiency of Inference Algorithms for Clinical Laboratory Data Sets: Green Artificial Intelligence Study
Source: J Med Internet Res. 2022 Jan 25;24(1):e28036. doi: 10.2196/28036 (PMC8826151; doi:10.2196/28036)
Supplement: Multimedia Appendix 1 [file jmir_v24i1e28036_app1.docx]

**Multimedia Appendix 1.** Time complexity of some common algorithms. The time complexity was estimated from the number of elementary operations, and it was in an asymptomatic expression because determining the exact number of operations was complicated. For instance, the complexity of LR increased with the number of features (*P*). The complexity of the algorithms was also related to the number of training set cases, number of decision trees (*T*), average depth of the decision trees (*D*), number of support vectors (*S*), and number of hidden units in a specific NN layer (*R*).

| Algorithm | Time complexity | Note |
| --- | --- | --- |
| Logistic regression | $O(P)$ |  |
| k-Nearest neighbors[1] | $O(PN)$ | Complexity without data preprocessing |
| Support vector machine[2] | $O(PS)$ | Complexity of a radial basis function kernelized SVM |
| Random forest[3] | $O(TD)$ |  |
| Extreme Gradient boosting | $O(TD)$ |  |
| Neural network | $O\left( \sum_{i=1}^{L} R_{i-1}R_{i} \right)$ | Complexity in a fullyconnected layer |

**References**

1. Kibriya AM, Frank E. An empirical comparison of exact nearest neighbour algorithms. European Conference on Principles of Data Mining and Knowledge Discovery. 2007:140-51.

2. Murphy KP. Machine learning: a probabilistic perspective: MIT press; 2012. ISBN: 0262304325.

3. Louppe G. Understanding random forests: From theory to practice. arXiv preprint arXiv:14077502. 2014.
