# Supplementary material for: Energy Efficiency of Inference Algorithms for Clinical Laboratory Data Sets: Green Artificial Intelligence Study
Source: J Med Internet Res. 2022 Jan 25;24(1):e28036. doi: 10.2196/28036 (PMC8826151; doi:10.2196/28036)
Supplement: Multimedia Appendix 2 [file jmir_v24i1e28036_app2.docx]

**Multimedia Appendix 2.** Classification performance of nonneural network-based ML algorithms implemented on the Mass Spectrometry and Urinalysis datasets. Accuracy and AUROC are presented with a 95% confidence interval. LR, logistic regression; kNN, k-nearest neighbors; SVM, support vector machine; RF, random forest; XGB, extreme gradient boosting; AUROC, area under the receiver operating characteristic.

| Dataset | Algorithm | Accuracy (%) | AUROC (%) |
| --- | --- | --- | --- |
| Mass Spectrometry | LR | 73.6 (71.4-75.8) | 80.8 (78.7-83.0) |
|  | kNN | 68.3 (66.0-70.6) | 74.7 (72.3-77.1) |
|  | SVM | 74.5 (72.3-76.7) | 81.3 (79.2-83.5) |
|  | RF | 77.5 (75.4-79.6) | 84.7 (82.8-86.6) |
|  | XGB | 77.2 (75.1-79.3) | 83.9 (82.0-85.9) |
| Urinalysis | LR | 79.1 (78.9-79.2) | 87.8 (84.9-90.5) |
|  | kNN | 74.9 (74.7-75.1) | 85.6 (82.8-88.3) |
|  | SVM | 69.2 (69.1-69.4) | 81.7 (78.9-84.4) |
|  | RF | 80.2 (80.1-80.4) | 91.1 (89.3-92.9) |
|  | XGB | 80.2 (80.1-80.4) | 91.4 (89.6-93.2) |
