# Supplementary material for: Energy Efficiency of Inference Algorithms for Clinical Laboratory Data Sets: Green Artificial Intelligence Study
Source: J Med Internet Res. 2022 Jan 25;24(1):e28036. doi: 10.2196/28036 (PMC8826151; doi:10.2196/28036)
Supplement: Multimedia Appendix 3 [file jmir_v24i1e28036_app3.docx]

**Multimedia Appendix 3.** Classification performance of different NNs implemented on the Mass Spectrometry and Urinalysis datasets. Accuracy and AUROC are presented with a 95% confidence interval. NN1, one-hidden-layer neural network; NN5, five-hidden-layer neural network; AUROC, area under the receiver operating characteristic.

| Dataset | Algorithm | Accuracy (%) | AUROC (%) |
| --- | --- | --- | --- |
| Mass Spectrometry | NN1 | 74.0 (71.8-76.2) | 80.6 (78.5-82.8) |
|  | Quantized NN1 | 73.3 (71.1-75.5) | 80.6 (78.5-82.8) |
|  | Pruned NN1 with 25% sparsity | 73.2 (70.9-75.4) | 80.6 (78.5-82.8) |
|  | Pruned NN1 with 50% sparsity | 73.9 (71.7-76.1) | 80.9 (78.8-83.1) |
|  | Pruned NN1 with 75% sparsity | 75.1 (72.9-77.2) | 80.8 (78.6-83.0) |
|  | NN5 | 75.7 (73.5-77.8) | 81.0 (78.9-83.2) |
|  | Quantized NN5 | 75.7 (73.5-77.8) | 80.6 (78.5-82.8) |
|  | Pruned NN5 with 25% sparsity | 75.5 (73.3-77.6) | 81.0 (78.8-83.2) |
|  | Pruned NN5 with 50% sparsity | 75.7 (73.5-77.8) | 81.1 (79.0-83.3) |
|  | Pruned NN5 with 75% sparsity | 74.9 (72.8-77.1) | 80.8 (78.6-83.0) |
| Urinalysis | NN1 | 79.5 (79.4-79.7) | 90.6 (88.8-92.5) |
|  | Quantized NN1 | 73.4 (73.3-73.6) | 88.6 (86.6-90.6) |
|  | Pruned NN1 with 25% sparsity | 75.4 (75.3-75.6) | 87.9 (86.0-89.9) |
|  | Pruned NN1 with 50% sparsity | 75.4 (75.3-75.6) | 87.9 (86.0-89.9) |
|  | Pruned NN1 with 75% sparsity | 74.4 (74.2-74.6) | 84.7 (82.5-87.0) |
|  | NN5 | 83.1 (83.0-83.3) | 84.4 (81.6-87.2) |
|  | Quantized NN5 | 73.4 (73.2-73.5) | 88.6 (86.6-90.6) |
|  | Pruned NN5 with 25% sparsity | 80.0 (79.8-80.2) | 82.9 (79.8-85.9) |
|  | Pruned NN5 with 50% sparsity | 85.8 (85.7-85.9) | 81.0 (77.4-84.7) |
|  | Pruned NN5 with 75% sparsity | 78.9 (0.78.7-79.1) | 74.5 (70.6-78.4) |
