# Supplementary material for: Energy Efficiency of Inference Algorithms for Clinical Laboratory Data Sets: Green Artificial Intelligence Study
Source: J Med Internet Res. 2022 Jan 25;24(1):e28036. doi: 10.2196/28036 (PMC8826151; doi:10.2196/28036)
Supplement: Multimedia Appendix 4 [file jmir_v24i1e28036_app4.docx]

**Multimedia Appendix 4.** Inferencing time and average power consumption levels of nonneural network-based algorithms implemented on the Mass Spectrometry and Urinalysis datasets. The results are presented as medians with the 25th and 75th percentiles. LR, logistic regression; kNN, k-nearest neighbors; SVM, support vector machine; RF, random forest; XGB, extreme gradient boosting.

| Dataset | Algorithm | Time (ms) | Average power (W) |
| --- | --- | --- | --- |
| Mass Spectrometry | LR | 0.47 (0.47-0.47) | 10.48 (10.35-10.72) |
|  | kNN | 0.55 (0.55-0.59) | 12.55 (11.99-12.69) |
|  | SVM | 0.90 (0.89-0.92) | 9.63 (9.43-9.82) |
|  | RF | 0.85 (0.82-0.87) | 9.93 (9.72-10.17) |
|  | XGB | 0.47 (0.47-0.47) | 9.42 (9.30-9.66) |
| Urinalysis | LR | 0.46 (0.45-0.46) | 9.98 (9.87-10.14) |
|  | kNN | 0.54 (0.53-0.55) | 11.41 (11.18-11.69) |
|  | SVM | 0.49 (0.46-0.49) | 10.13 (9.93-10.54) |
|  | RF | 0.97 (0.95-0.99) | 10.33 (10.17-10.52) |
|  | XGB | 0.39 (0.39-0.40) | 10.43 (10.23-10.54) |
