# Supplementary material for: Energy Efficiency of Inference Algorithms for Clinical Laboratory Data Sets: Green Artificial Intelligence Study
Source: J Med Internet Res. 2022 Jan 25;24(1):e28036. doi: 10.2196/28036 (PMC8826151; doi:10.2196/28036)
Supplement: Multimedia Appendix 5 [file jmir_v24i1e28036_app5.docx]

**Multimedia Appendix 5.** Inferencing time and average power consumption levels of different NNs implemented on the Mass Spectrometry and Urinalysis datasets. The results are presented as medians with the 25th and 75th percentiles. NN1, one-hidden-layer neural network; NN5, five-hidden-layer neural network.

| Dataset | Algorithm | Time (ms) | Average power (W) |
| --- | --- | --- | --- |
| Mass Spectrometry | NN1 | 0.61 (0.57-0.61) | 11.07 (10.81-11.80) |
|  | Quantized NN1 | 0.60 (0.56-0.61) | 10.81 (10.81-11.80) |
|  | Pruned NN1 with 25% sparsity | 0.60 (0.55-0.61) | 10.84 (10.59-11.68) |
|  | Pruned NN1 with 50% sparsity | 0.60 (0.54-0.61) | 11.59 (10.96-12.08) |
|  | Pruned NN1 with 75% sparsity | 0.60 (0.55-0.61) | 11.13 (10.90-11.94) |
|  | NN5 | 0.62 (0.61-0.63) | 12.42 (12.24-12.70) |
|  | Quantized NN5 | 0.61 (0.61-0.62) | 11.58 (11.40-11.76) |
|  | Pruned NN5 with 25% sparsity | 0.62 (0.61-0.63) | 12.87 (12.56-13.12) |
|  | Pruned NN5 with 50% sparsity | 0.62 (0.61-0.63) | 12.30 (12.04-12.50) |
|  | Pruned NN5 with 75% sparsity | 0.63 (0.62-0.64) | 12.37 (12.14-12.64) |
| Urinalysis | NN1 | 0.54 (0.51-0.55) | 11.45 (10.86-11.74) |
|  | Quantized NN1 | 0.53 (0.51-0.55) | 11.03 (10.59-11.32) |
|  | Pruned NN1 with 25% sparsity | 0.52 (0.51-0.55) | 11.38 (10.89-11.59) |
|  | Pruned NN1 with 50% sparsity | 0.53 (0.51-0.55) | 10.81 (10.49-11.20) |
|  | Pruned NN1 with 75% sparsity | 0.53 (0.52-0.55) | 10.90 (10.47-11.15) |
|  | NN5 | 0.58 (0.55-0.59) | 11.46 (11.07-11.94) |
|  | Quantized NN5 | 0.58 (0.55-0.59) | 11.16 (10.78-10.68) |
|  | Pruned NN5 with 25% sparsity | 0.59 (0.56-0.59) | 11.59 (11.38-12.17) |
|  | Pruned NN5 with 50% sparsity | 0.58 (0.55-0.59) | 11.29 (11.04-11.87) |
|  | Pruned NN5 with 75% sparsity | 0.58 (0.55-0.59) | 10.74 (10.48-11.32) |
