# Supplementary material for: Energy Efficiency of Inference Algorithms for Clinical Laboratory Data Sets: Green Artificial Intelligence Study
Source: J Med Internet Res. 2022 Jan 25;24(1):e28036. doi: 10.2196/28036 (PMC8826151; doi:10.2196/28036)
Supplement: Multimedia Appendix 7 [file jmir_v24i1e28036_app7.docx]

**Multimedia Appendix 7.** *P* values were derived from the pairwise Wilcoxon signed-rank test to identify which time and power consumption of any two algorithms were different on the Urinalysis dataset. The adjusted *P* values was adjusted by the Bonferroni multiple testing correction method. LR, logistic regression; kNN, k-nearest neighbors; SVM, support vector machine; RF, random forest; XGB, extreme gradient boosting; NN1, one-hidden-layer neural network; QNN, quantized five-hidden-layer neural network; PNN, pruned five-hidden-layer neural network; NN5, five-hidden-layer neural network.

|  | | Time consumption | | Power consumption | |
| --- | --- | --- | --- | --- | --- |
| Algorithm | | *P* value | Adjusted *P* value | *P* value | Adjusted *P* value |
| LR | kNN | <.001 | <.001 | <.001 | <.001 |
| LR | SVM | <.001 | <.001 | .003 | .06 |
| LR | RF | <.001 | <.001 | <.001 | <.001 |
| LR | XGB | <.001 | <.001 | <.001 | .008 |
| LR | NN1 | <.001 | <.001 | <.001 | <.001 |
| LR | NN5 | <.001 | <.001 | <.001 | <.001 |
| LR | QNN | <.001 | <.001 | <.001 | <.001 |
| LR | PNN | <.001 | <.001 | <.001 | <.001 |
| kNN | SVM | <.001 | <.001 | <.001 | <.001 |
| kNN | RF | <.001 | <.001 | <.001 | <.001 |
| kNN | XGB | <.001 | <.001 | <.001 | <.001 |
| kNN | NN1 | <.001 | <.001 | <.001 | <.001 |
| kNN | NN5 | <.001 | <.001 | <.001 | <.001 |
| kNN | QNN | <.001 | <.001 | .003 | .10 |
| kNN | PNN | <.001 | <.001 | .42 | 1 |
| SVM | RF | <.001 | <.001 | .04 | .93 |
| SVM | XGB | <.001 | <.001 | .18 | 1 |
| SVM | NN1 | <.001 | <.001 | <.001 | <.001 |
| SVM | NN5 | .36 | 1 | <.001 | <.001 |
| SVM | QNN | <.001 | <.001 | <.001 | <.001 |
| SVM | PNN | <.001 | <.001 | <.001 | <.001 |
| RF | XGB | <.001 | <.001 | .83 | 1 |
| RF | NN1 | <.001 | <.001 | <.001 | <.001 |
| RF | NN5 | <.001 | <.001 | <.001 | <.001 |
| RF | QNN | <.001 | <.001 | <.001 | <.001 |
| RF | PNN | <.001 | <.001 | <.001 | <.001 |
| XGB | NN1 | <.001 | .001 | <.001 | .001 |
| XGB | NN5 | <.001 | <.001 | <.001 | <.001 |
| XGB | QNN | <.001 | <.001 | <.001 | <.001 |
| XGB | PNN | <.001 | <.001 | <.001 | <.001 |
| NN1 | NN5 | <.001 | <.001 | <.001 | <.001 |
| NN1 | QNN | <.001 | <.001 | .31 | 1 |
| NN1 | PNN | <.001 | <.001 | .61 | 1 |
| NN5 | QNN | .07 | 1 | <.001 | .03 |
| NN5 | PNN | .54 | 1 | .30 | 1 |
| QNN | PNN | .25 | 1 | .06 | 1 |
